# Social Representations and Practices Towards Triatomines and Chagas Disease in Calakmul, México

**DOI:** 10.1371/journal.pone.0132830

**Published:** 2015-07-23

**Authors:** Alba Valdez-Tah, Laura Huicochea-Gómez, Judith Ortega-Canto, Austreberta Nazar-Beutelspacher, Janine M. Ramsey

**Affiliations:** 1 Departamento de Sociedad y Cultura, El Colegio de la Frontera Sur, Campeche, Campeche, México; 2 Centro Regional de Investigación en Salud Pública, Instituto Nacional de Salud Pública, Tapachula, Chiapas, México; 3 Centro Regional de Investigaciones Biomédicas “Dr. Hideyo Noguchi”, Universidad Autónoma de Yucatán, Mérida, Yucatán, México; 4 Departamento de Salud, El Colegio de la Frontera Sur, San Cristóbal de las Casas, Chiapas, México; Université de Perpignan Via Domitia, FRANCE

## Abstract

Vector-borne transmission of *Trypanosoma cruzi* (VBT*Tc*) is dependent on the concomitant interaction between biological and environmental hazard over the entire landscape, and human vulnerability. Representations and practices of health-disease-care-seeking and territorial appropriation and use were analyzed for VBT*Tc* in a qualitative ethnographic study in the Zoh-Laguna landscape, Campeche, Mexico. In-depth interviews and participatory observation explored representations and practices regarding ethno-ecological knowledge related to vector-transmission, health-disease-care-seeking, and land use processes. The population has a broad knowledge of biting insects, which they believe are all most abundant in the rainy season; the community´s proximity to natural areas is perceived as a barrier to control their abundance. Triatomines are mostly recognized by men, who have detailed knowledge regarding their occurrence and association with mammals in non-domestic fragments, where they report being bitten. Women emphasize the dermal consequences of triatomine bites, but have little knowledge about the disease. Triatomine bites and the *chinchoma* are “normalized” events which are treated using home remedies, if at all. The neglected condition of Chagas disease in Mexican public health policies, livelihoods which are dependent on primary production, and gender-related knowledge (or lack thereof) are structural circumstances which influence the environment and inhabitants´ living conditions; in turn, these trigger triatomine-human contact. The most important landscape practices producing vulnerability are the activities and mobility within and between landscape fragments causing greater exposure of inhabitants primarily in the dry season. A landscape approach to understanding vulnerability components of VBT*Tc* from health-disease-care-seeking perspectives and based on territorial appropriation and use, is essential where there is continuous movement of vectors between and within all habitats. An understanding of the structural factors which motivate the population´s perceptions, beliefs, and practices and which create and maintain vulnerability is essential to develop culturally relevant and sustainable community-based VBT*Tc* prevention and control.

## Introduction

Human vulnerability to vector-borne transmission of *Trypanosoma cruzi* (VBT*Tc*), etiologic agent of Chagas disease (CD), occurs as a result of practices related to triatomine vector exposure over the entire landscape, and not only in domestic fragments [1, 2]. Vector-borne transmission of *T*. *cruzi* occurs only in the American continent where 8–10 million people are affected by the disease and close to 25 million are at risk for infection. Chagas disease is one of the most important parasitic disease in Mexico as in the Americas, where inhabitants principally become infected when the vector defecates during its blood meal [3–6]. The parasite contained in fecal material penetrates through the bite wound or mucous membranes into the circulatory system and internal organs [3]. Under the assumption that vector-human contact principally occurs inside domiciles, CD control programs have historically only targeted domesticated triatomine populations for reduction using large-scale application of residual insecticides [1]. However, human exposure to VBT*Tc* occurs across the landscape, in both conserved and modified habitats to differing degrees, by almost all vector species, principally due to human ecosystem modification and use, the biology and ecology of triatomines and their hosts, features particular to human housing which are advantageous for bug fitness, and the opportunistic use of humans as blood-source [2]. These hazard components provide the conditions for human exposure which, concomitant with contact vulnerability in all habitat fragments (domestic, ecotone, and sylvatic), produce transmission risk for VBT*Tc* [1, 2, 7]. Deforestation and modification of the landscape affect the composition and diversity of *T*. *cruzi* reservoir communities and infected vectors, and VBT*Tc* increases when human populations are not protected against this exposure [8–12]. As long as landscapes continue to be modified, human exposure to triatomines will occur if vulnerability in all habitats is not reduced [1, 8]. Transmission of *T*. *cruzi* to humans not only depends on the spatial characteristics of reservoirs, humans, vector, and parasite populations, but also on their temporal dynamics in all landscape fragments.

House infestation and re-infestation after domestic triatomine control is principally due to the presence of infected wildlife, livestock, and pets in and around houses, and the physical conditions and maintenance practices for houses and immediate surroundings [13–18]. These and other factors such as inhabitants´ occupation, cultural practices, livestock confinement practices, household economy, and social priorities have highlighted how the diversity of human practices and sociocultural context affect VBT*Tc* [14, 19–22]. These same sociocultural factors have an impact on human movement, reservoir communities, and their interactions within the landscape and between habitats, all fundamental for vector and pathogen dispersal. However, most triatomine control initiatives have focused uniquely on domestic vector populations since these were obvious primary targets for control, despite evidence that vector transmission of *T*. *cruzi* to humans also occurs in extra-domestic spaces. Most vector species have continuous populations throughout the landscape, and vectors have different degrees of contact with humans in different habitats, even though few studies have measured the proportional contribution of vector transmission from extra-domestic interactions [2, 8, 11].

Sociocultural and economic factors which expose populations to vectors and result in CD risk have been described in Venezuela, Brazil, and Argentina [23, 24]. In these studies, lack of land tenure was shown to impede the construction of more solid housing and affects the population´s choice of construction materials and methods [23]. Failure of Argentina’s vector control activities in the Gran Chaco region has been attributed to conceptual differences between prevention program and target populations regarding ‘‘landscape”, ‘‘wild”, and ‘‘domesticated” [24]. Local populations conceive and use “peridomestic” areas as an extension of the domestic as well as other habitats, thereby permitting movement of humans, animals, and plants from the mountain or agricultural areas to ‘‘domestic” spaces. However, prevention programs consider extra-domestic spaces separate, in order to focus on interventions only in houses. Analysis of structural (sociocultural, political, economic, historical) circumstances which influence living conditions and the inhabitant´s use of the landscape is crucial to understanding vulnerability for human-triatomine interactions, and VBT*Tc*.

Although sociocultural and economic aspects are recognized determinants of human vulnerability for VBT*Tc* and CD, social science and qualitative research contributions to this field of study are scarce [25–28]. Most studies focus on using rapid assessment of knowledge, attitudes, and practices (KAP) in order to generate information regarding what people know or say they do, which may vary considerably from their behavior, and provides no information on underlying structural living conditions and social representations, such as health care access, livelihoods, ecological settings, and historical processes [27]. As a result, public healthcare programs assume that if the population has more biomedical information, this alone will convert to knowledge and motivate prevention and control behavior, which is usually not the case [25]. Information from rapid assessments is insufficient and cannot substitute ethnographic fieldwork to analyze associations from the exposed population´s reality [27, 28]. An analysis of structural determinants and specific practices related to human interactions within landscapes and to health-disease-care-seeking processes are essential in order to gain a better understanding of vector-borne disease epidemiology [2].

Studies on disease risk perception according to inhabitants mindset, how they develop opinions, attitudes, beliefs, and values, have demonstrated that all affect and explain behaviors related to vector control, prevention and healthcare seeking practices [25, 29–43]. In some endemic areas, CD has been naturalized and normalized to the point that it is not perceived as life threatening or a health priority [29, 31, 34, 35, 41]. In some cultures, CD is thought to be provoked by the individual, or as a result of violating taboos or social norms [29, 34, 35, 37]. Vectors are bearers of good luck in some indigenous Bolivian beliefs, and in Mexico they have aphrodisiac and culinary uses, while in Brazil they have therapeutic properties [40, 44–46]. Preventive and curative practices related to vector-borne diseases (VBD) are, therefore, deeply linked to sociocultural representations, rather than the presence of a parasite in an insect vector [25].

The population´s understanding of risk, and the social, economic, and historical factors that influence their practices and living conditions are essential for human vulnerability to vector-borne disease transmission, including VBT*Tc* [2, 25, 47–49]. Most interventions do not integrally analyze or address these elements, or adopt participatory approaches by incorporating local perspectives and experiences into prevention, surveillance, and control programs. In order to appropriately characterize VBT*Tc* risk, socio-cultural practices and representations, factors and processes that increase human vulnerability to infection in different socio-environmental contexts, are fundamental evidence to design effective strategies, and adapt interventions to local settings.

*Triatoma dimidiata* is the only *T*. *cruzi* vector reported in Campeche, and particularly surrounding Zoh-Laguna, embedded in the Calakmul.Biosphere Reserve. Results from a simultaneous study demonstrate an estimated 3% human prevalence in this community, abundance of natural and alternative *T*. *cruzi* reservoirs, and continuous parasite transmission across conserved and modified habitats. Greatest human contact to the bugs in sylvatic and ecotone habitats occurs at the end of the dry season, when seasonal reduction of natural reservoir abundance motivate alternative host-seeking activity, even though infected bugs maintain uniform year-round resource-seeking activity, colonization, and infestation in and around houses (domestic habitat) [50]. The population reports contact with bugs in all landscape fragments, resulting in variable-sized *chinchomas* for some inhabitants (induration due to salivary proteins), while others report no remarkable reaction. The primary aims of the present study were to characterize and link social representations and practices of health-disease-care-seeking and territorial appropriation processes and their structural determinants to exposure for VBT*Tc* in Zoh-Laguna, Campeche. Principal dimensions of daily-life and significance structures which guide behavior and provide the opportunity for effective social communication and comprehension of the material and social environment were analyzed in relation to vector exposure and persistence across the landscape, and CD prevention.

## Materials and Methods

### Ethics statement

The research protocol for this study was approved by the Ethics Committee Review Board of the National Institute of Public Health of Mexico. County and community authorities were first approached to explain the study purpose, for which they gave their verbal collective consent. All participants were informed of the study´s purpose and that they could freely participate or quit the study at any time. They gave their written consent before data collection (interview recording, field-notes, and photographs) and received contact information for the research team and review board for further queries or comments regarding their participation.

### Study setting: Zoh-Laguna landscape and inhabitants

**Fig 1:**
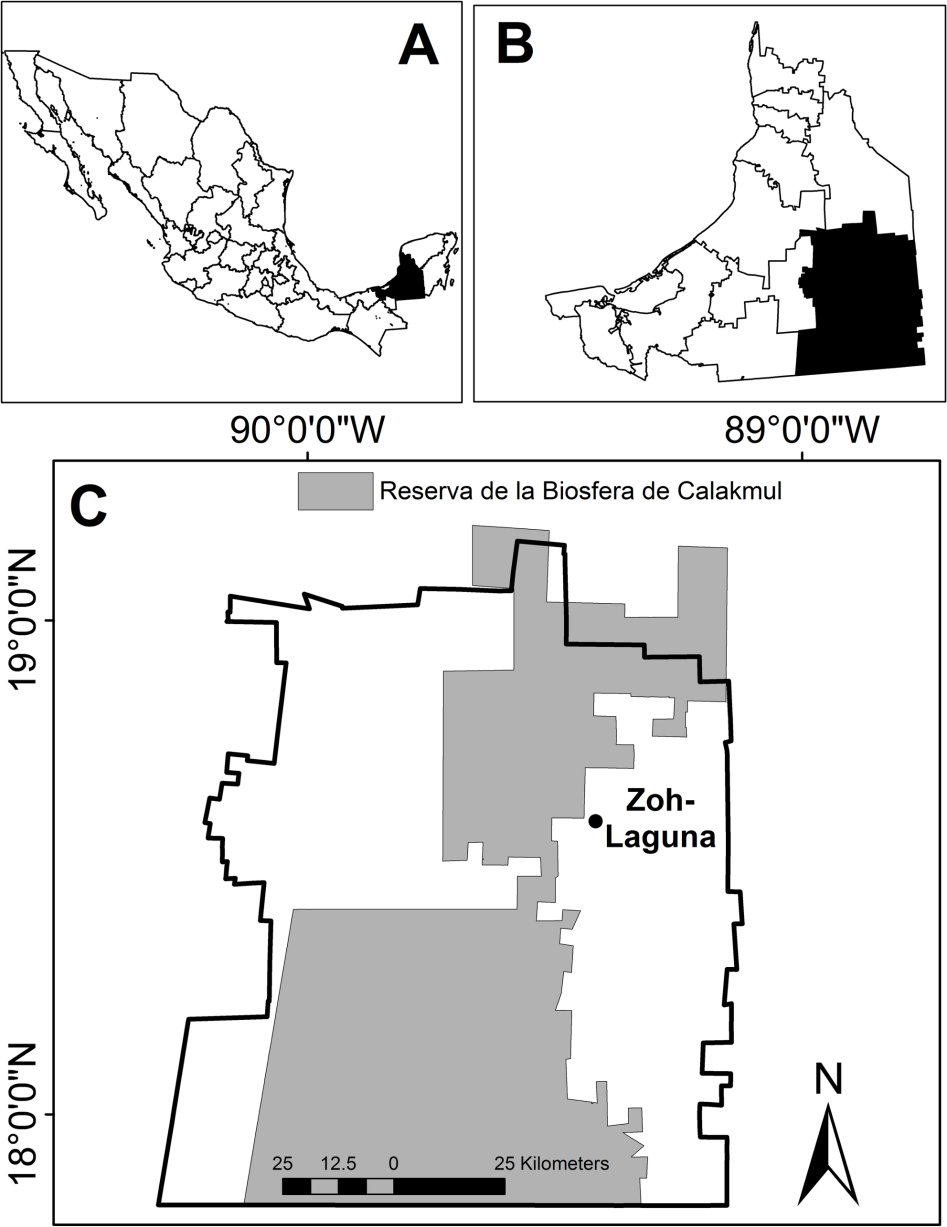
Map of Zoh-Laguna, Calakmul, Campeche, México. A: Campeche State in Mexico. B: Calakmul County. C: Zoh-Laguna and Calakmul Biosphere Reserve.

Zoh-Laguna is located in Calakmul county of Campeche, in southeast Mexico (Fig 1). The current community was founded as a timber camp in the 1950’s and is the oldest modern community in the region with 1,074 inhabitants [51]. Current inhabitants are originally from other regions of the Yucatan Peninsula (some of them are maya-speakers), with cho’ol and tzeltal ethnic groups from Chiapas who settled in the 1980’s. Additional immigrants from south and central Mexico integrated into the community in the last decade. The residential area and 70,000 surrounding hectares are *ejido* land, an agrarian law for collective communal property that preserves and regulates the collective holding, access, and use of land in Mexico [51]. There are 135 *ejidatarios* (93.8% men) in the assembly who decide and manage all land use practices and related activities. Families living in the community who are not *ejidatarios* are designated as rentors (“pobladores”; 121/256), some of whom are owners of recently privatized land.

**Fig 2:**
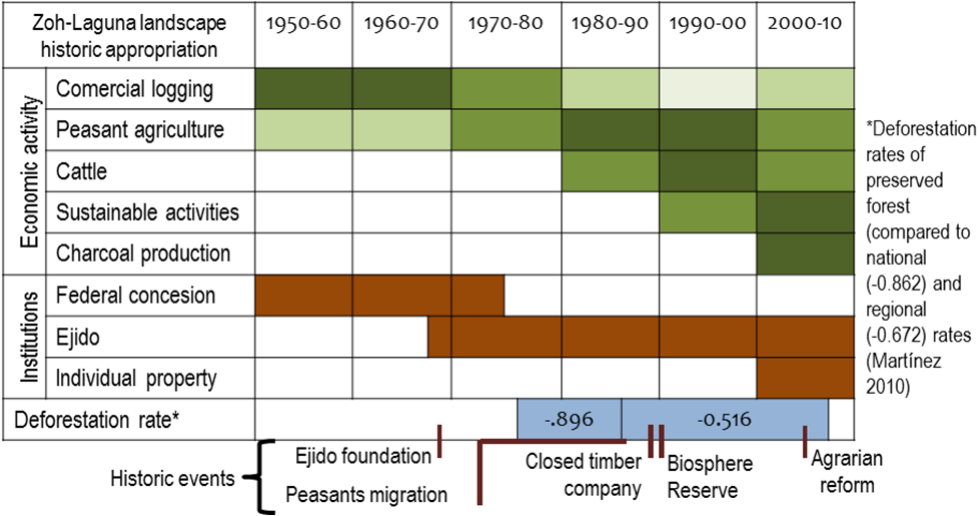
Historical social appropriation of the Zoh-Laguna landscape based on economic activities and social structures in the last half of the twentieth century to the present day. The intensity of colors in the matrix indicates the intensity of the economic activity: darker hues indicate most intensive activity, and *vice versa*.

Primary productive activities in the landscape are charcoal production, agriculture, timber extraction, reforestation and conservation projects, small scale livestock rearing, and hunting. Historical land use transformation and collective or private land tenure define the current spatial configuration of the Zoh-Laguna landscape (Fig 2). Agrarian regulation and environmental protection in recent decades have contributed to local awareness for forest conservation and diversification of primary economic activities and family income sources are focused on the *acahual* (farming plots) (Table 1).

**Table 1:** Definition and characteristics of habitats according to local knowledge and meanings (and nearest English translation) of terms used by participants for the different fragments of the landscape.

Term and equivalent definition	Characteristics of habitats
“Montaña”. Natural forested areas, conserved; sylvatic habitat.)	- Original forest, which has high social value aesthetically and for emotional reasons, due to natural products and wildlife (bushmeat, hard wood, gum resin, orchids),
	- Considered a pristine habitat, with high canopy, clean and shaded paths highly valued
	- Narrations about this habitat are nostalgic based on memories from inhabitant´s youth (older inhabitants) and former labor activities with gum resin and lumber extraction.
“Acahual”. Farming plots; (Ecotone)	- Grassland areas with primary or secondary growth or brush, either abandoned or managed through multiple slash- burn agriculture cycles.
	- Habitat for many economic activities: milpa, crops, charcoal production, firewood collection, grass cultivation, pastureland, and apiculture.
“Espacio *lóbrigo”*. Overgrown areas in the community; domestic space.	- Rustic areas within or surrounding community with unwanted vegetation and undergrowth.
	- It is considered wild and “undomesticated” vegetation, unkept and dirty where harmful poisonous animals and insects hide.
“Monte”. Grasses, undergrowth.	- Vegetation that grows without human intervention, associated with domiciles and unkept community areas.
	- Monte refers to the vegetation found in different areas: *acahual*, *montaña* and the “espacios lóbrigos”. Each area has different uses and is valued differently by the population.

The town of Zoh-Laguna has 265 dwellings with highest density in its center [51]. Wood walls, zinc roofs, and concrete floors are the most common housing characteristics (67%), with remaining houses made of brick or cement blocks. Houses are surrounded by variable-sized peridomiciles (1200–1400 m^2^) used for domestic practices, social interactions, and family activities such as cooking, laundry, animal confinement, natural and agricultural product storage, fruit trees, vegetable, medical, and ornamental plant cultivation, as well as latrines.

A primary care medical unit from the Instituto Mexicano del Seguro Social—Prospera (IMSS-Prospera, formerly IMSS-Oportunidades; www.imss.gob.mx/imss-prospera) is located in Zoh-Laguna, and a second level primary healthcare hospital from the Secretaria de Salud de Campeche (SSC) is located 10km from the town, in Xpujil. More than half of the families (135/256) receive Prospera subsidy and nutritional supplements (http://www.sedesol.gob.mx/es/SEDESOL/Prospera). As part of this program, the female head of household is required to participate in health talks, workshops, and backyard and public area “cleaning” activities. All family members must commit to attend programmed health consults for priority programs (i.e. vaccination among others). Community leaders (*ejidatarios*), women, men, and elementary school children of Zoh-Laguna were initially engaged with information about CD and vector-transmission in order to obtain approval to conduct in-depth landscape studies [50]. Inhabitants and a group of children and young adult volunteers were trained to find and collect vectors in houses and community spaces, while men provided information regarding the location and abundance of small and medium-sized mammals in the landscape. No previous studies regarding CD had been conducted in the town or greater landscape either by academic groups or healthcare personnel.

### Study participants

Participants were selected using an intentional sampling process that focused on sociocultural representations [52]. An exploratory study [2] identified inhabitants who complied with two or more of the following inclusion criteria: adult (women or men) with children of school age, individuals who work in one of the population´s principal economic activities (milpa, charcoal production, livestock production, or beekeeping), either *ejidatario* or *poblador*, and registered in the Prospera program. These inhabitants were invited to participate in activities conducted in their domiciles.

### Data collection and analysis

Ethnographic data were collected over 10 months between 2011 and 2012 from 22 in-depth interviews. The total number of interviews was defined by theoretical saturation, terminating when no new information or data appear from each additional respondent, and conceptual insights are well-developed. [53, 54]. Participant observation was conducted by a professional anthropologist (AVT) who had participated previously in CD information and engagement activities, and who has social experience and knowledge regarding local culture and language. Interview topics are listed in Table 2, and were completed in two visits with each participant during their work activities. Digital audio recordings from interviews were transcribed to a Word text program. Eight additional semi-structured interviews were applied to persons with the greatest knowledge and experience in principal economic activities (farmers, charcoal producers, cattle rancher, and beekeeper), to inquire about land use, modification practices, and movement between habitats. These latter interviews were only recorded using field-notes. An annual activities calendar was developed to summarize the population´s spatial and seasonal activities.

**Table 2:** Deep interview topics regarding human vulnerability components for VBT*Tc* from health-disease-care-seeking and land appropriation and use landscape frameworks in Zoh-Laguna.

Exposure hazard	Health, disease and care-seeking processes	Land appropiation and use
1.Ecosystem modification and use	The relationship between 1) ecosystem use and modification, and 2) impact of movement between habitats for health and disease	Spatial and temporal dynamics of human practices in the ecosystem-landscape, and their effects on wildlife and insects
2. Triatomines as vectors	Insects and triatomines in human health: disease and dermal consequences, pathogen transmission mechanisms, susceptibility for bites, treatment, and prevention practices, interactions with animals and related practices.	Insect biology and behavior and infection with disease agents; landscape localization; interaction with mammals, insect control practices; livestock management and practices, and their potential infection.
3. Human domestic habitat	Internal house structure and organization, the peridomicile, relationship of spaces with health-disease and human practices that affect either.	House construction, arrangement of spaces, connection with other habitats; materials storage and accumulation; house’ penetrability and stability for triatomines
4. Humans as a triatomine host	Sleep practices and the perception of protection/threat during sleep periods from triatomines (day or night).	Sleep practices in the different habitats (seasonality, according to age, sex, and occupation), and perception of protection or threat in extra-domestic habitats.

Participant observation was conducted with men during their workday in field activities (when they were accompanied by their spouse or another relative), with women during their activities around the community, with neighbors in their *milpa*, in the domestic spaces of five families, in occasional visits to other households, and during collective community activities. The themes registered from participant observation are listed in Table 2. Aspects of daily-life related to local experiences which contextualized what people say versus what they do, were also registered. Data collection, informal conversations, observed practices, and field processes were registered in a field journal.

Fieldwork notes and transcript interviews were analyzed in an inductive way by AVT according to the primary themes listed in Table 2, but not constrained by concepts derived from the research topics [52, 53]. The analysis identified emerging categories by re-reading and coding field notes, without qualitative software text-analysis. Once coded, data were compiled and analyzed for their characteristics and meanings. Data analysis also used a verification technique for what people say and do using interview and participant observation data as independent sources. This method identified commonalities and differences in practices and meanings [54]. Themes and sub-themes were cross-checked within the situational knowledge provided by long term participation, observation, and relationships. Representative anonymized quotes from interview transcripts are included in the results and observations from field notes are reported by location, field note page number, and date (e.g. household pp. 13–14, 02/11/07).

## Results

### A dual and antagonistic relationship between environment and human health

The majority of participants had dual and antagonistic perceptions related to the environment and human health. Generally, the *monte* (Table 1) and trees were mentioned frequently as sources of “fresh air”, important for breathing and to help dissipate heat. Hence, deforestation was considered negative since it reduces air quality and causes higher ambient temperature. These latter conditions are perceived to cause flu, cough, and diarrhea. The positive value of the *monte*, however, was contrasted with it being a shelter for dangerous animals and insects, harmful to human health. Animals mentioned were snakes, the *mosca chiclera* (phlebotomine sandflies), and *el tigre* (wild felines). An overriding threat to the population and their health from the *monte* was mentioned by some participants as the reason why all natural vegetation surrounding dwellings and the town should be eliminated.

### “Normalization” of insect and triatomine bites

**Fig 3:**
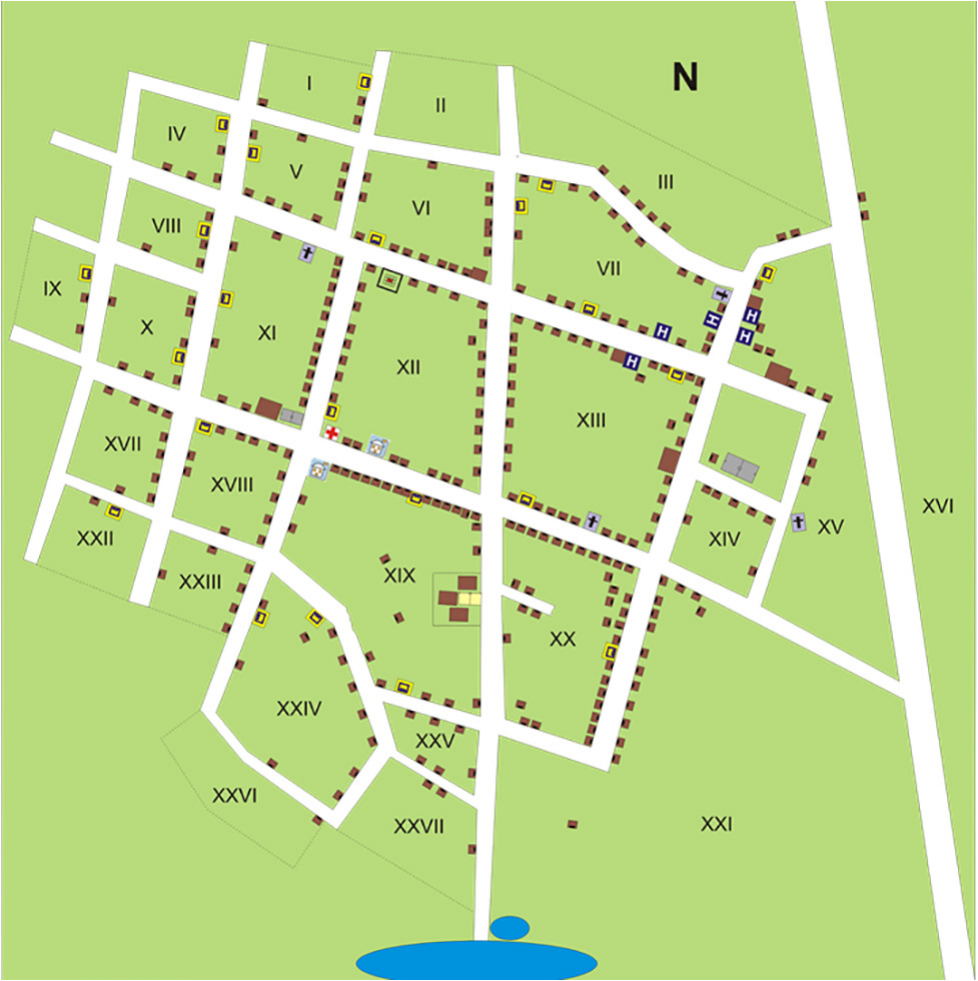
Map of Zoh-Laguna with approximate location (to guarantee anonymity) of each participant´s house.

**Fig 4:**
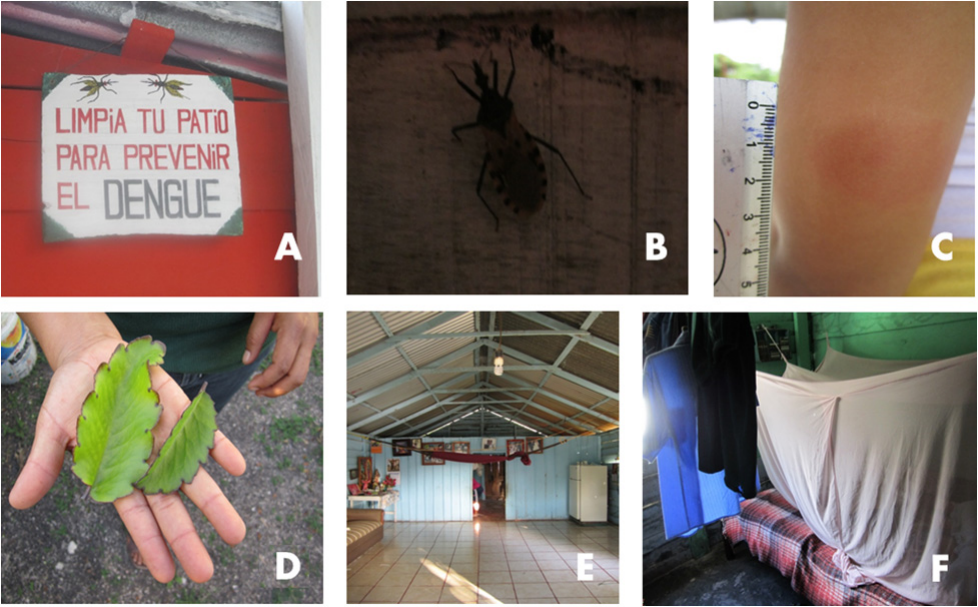
Images of health, disease, and care-seeking processes related to vector transmission of *Trypanosoma cruzi* in Zoh-Laguna, Calakmul. A: A sign elaborated by women from the Prospera program that exemplifies engagement for the “Patio limpio” (clean patio) dengue prevention program. B: Adult triatomine identified by inhabitants in a house on the periphery of the community. C: C*hinchoma* after 48 hrs from an eight year-old girl bitten inside her house. D: Belladona leaves used to treat *chinchoma* symptoms. F: Floor cleaning practices. E: Bednet use at night.

Many inhabitants reported multiple bites by different types of insects, referring to these as normal and daily events. A wound or swelling on the body is considered the result of an insect bite, although the population cannot always identify the insect responsible, nor can they recognize all insects, or the specific effect of each. Some mothers whose children had skin symptoms and wounds of unknown origin, questioned whether they might have been *chinchomas*, the term used for the inflammatory dermal process resulting from triatomine saliva (House #14, pag. 165–166, 12/04/2011 and 234, 167/01/2012; Fig 3). Both men and women cited a diversity of insects associated with disease and which are harmful to human health (Table 3). Mosquitoes and the *mosca chiclera* were the most frequent mentioned, as causing dengue, malaria, and the “*piquete de la mosca chiclera*” (cutaneous leishmanioses) (Fig 4A). Triatomines were mentioned by some individuals, although this was not consistent, and in the case of women from Prospera, may have resulted from recent information given to women through talks with the research project and associated with the interviewer.

**Table 3:** Information and knowledge regarding harmful insects and treatment practices reported by Zoh Laguna inhabitants. Insects are listed in order of frequency of being mentioned.

	Insect*	Affectation, harmful to health, source of disease	Origen/ cause	Infection/ contagious mechanism	Treatment practices	Preventive practices
1	Mosquitoes (*mosco*)	Infection, disease, flu, alergy, dengue, haemorrhagic dengue, malaria, hepatitis, dermal reactions (wheal, sore)	“Something”, infection, contaminated or bad blood, substance, virus	Bite, Sting	Alcohol, herbal ointment	Mosquito net, aerosol insecticides, repellent and fan
2	Gadfly (tábano)	Wheal, alergy, intoxication, throat closure, pain, swelling, fever	Poison, venom, contaminated blood	Sting, bite	Alcohol, herbal ointment, antihistamine (Avapena) injection	Repellent, none.
3	Phlebotomines (mosca chiclera)	Wheal, sore, skin cancer, wound, leishmaniosis, eats skin/flesh, painful, lethal.	Virus, venom, bad blood from biting other animals	Sting, bite	Biomedical treatment, boiled lemon with salt, plant *Xcanán*, *Chunub* tree resin	Thick clothing and long sleeve shirt, hats and gloves. It cannot be avoided
4	Cockroach	Wheal, alergy, petechia	Spittle, fungus	Bite, skin contact, sting	Wash with water and soap	Be wary
5	Scorpion	Pain, dizziness, intoxication, numbness, fever, wheal	Poison, toxin	Sting	Put something cold on the sting	Be wary
6	Bug (insectivorous, phytophagous)	Wheal, dermal irritation (stain, bulb, purple/ bruising, burning, burn)	Urine, poison, liquid, aroma, “*wix”*, substance, acid, venom	Skin contact, suck	Wash with water and soap, wipe with alcohol	Be wary
7	Venomous snake, (víbora)	Wound, sore	Poison	Sting, bite	Ties above the bite, needs medical attention, cheawing “contrahierba” plant	Be wary
8	Botfly (colmoyote)	Wheal, pain and swelling	Eggs	Bite	The larva has to be taken out from under the skin	It can be avoided, one doesn’t feel the insect
9	*Pic*, “las Chagas” (triatomine)	Wheal, wound, affects the heart, infarction, death	“Something”, parasite, poison, venom, eggs	Bite, excrement, pupu	Remedy belladonna plant, ointment and alcohol	Mosquito net, aerosol insecticides and fan
10	Tick (pech)	Rash, seizure disorder, sores, fever, skin diseases	Bite	Bite	Ointment and alcohol	Maintain animals outside and away from the house
11	Tarantula	Sores, irritation, allergy	Fur, lint	Skin contact	Wash with water and soap	Avoid touching the insects hairs
12	Spider (araña)	Infection of the skin, itching	Virus	Bite	Biomedical attention	None
13	Wasps (avispa)	Pain, fever, throat closure	Liquid	Bite	None	None
14	Beetle (carga- basura)	Wheal	Virus	Bite	None	None
15	Flea	Wheal, wound	Bite	Bite	Alcohol	Fumigate
16	Worm	Wheal, fever	Poison	Skin contact	Wash with water and soap, ointment	Avoid touching the insect

Knowledge and awareness of triatomines is heterogeneous among Zoh-Laguna inhabitants. They have seen them inside their houses or know them from the *monte*, using the Mayan name “*pic*”, although some of them recognize them only from health promotion images or because they have heard stories by other inhabitants, due to the concomitant Chagas transmission research project (Fig 4B). As a result of that project, the inhabitants referred popularly to triatomines as “la/las Chagas” (instead of “*pic*” or chinche, the latter is the common Spanish term). About one-third of those interviewed (7/22), either personally or from one of their family members, had reported a previous triatomine bite. In all cases, the bites were considered as normal events without perception of a threat at the time, occurring primarily but not exclusively inside houses. Participants pointed out that the first time they received information about triatomines as disease vectors was through the concomitant Chagas transmission research project (i.e. within the previous 2 yrs). Women from the Prospera program mentioned that triatomines “spread disease”, and focused on their blood-sucking behavior, and the *chinchoma* they provoked, and much less about the disease and its characteristics. In contrast, most men working in the *monte* did report knowing “*pic*”, but their relationship with a disease was even less mentioned. Men´s knowledge about the bugs was detailed, related to the landscape and the vector´s habits, encountered while hunting at night, camping in the forest, and siestas during work in the *monte*.

The *chinchoma* was described as hard “as a tumor”, with the form of “red wheal” that itches, swells, sometimes is even painful, feels hot, and “lasts for a long time”, from five to 14 days (Fig 4C). Two local concepts exist when referring to insect bites and their effect on the skin: *allergy* and *poison*. Both latter concepts are used by participants to explain why an insect produces different skin reactions or additional symptoms, dependent on the individual and/or on the nature of the insect. *Allergy* is referred as an innate physical condition or recurrent disease that predisposes individuals to other health problems. As a result, they become more vulnerable to situations that normally would not negatively affect their health. *Allergic* persons are considered weak and having fragile bodies, skin, and blood. This condition predisposes them to severe symptoms and abnormal reactions. When questioned about the duration of a mosquito bite, a woman stated:

F1: “It depends, sometimes it lasts over 2 or 3 days, but an allergic one can even produce pus” (housewife, 33 years-old).

In contrast, some participants, principally individuals working in the *monte* (men), considered that insect bites “don’t do anything to them”. They explain this lack of effect based on many previous bites experienced over their lifetime, which allows them to develop certain “resistance”. This self-perception of physical strength is illustrated by a farmer who reported several triatomine bites:

M1: “Well, we never were hurt by it [the triatomine], never, but as I repeat, one is already used to insect bites and all… well, they don’t do anything to us, but, if one is not used to them [to the triatomines bites] then they do hurt, they really hurt” (farmer, 65 years-old).

Some participants refer to insects carrying a *poison*, due to the symptoms after the bite:

M2: “Well, similar to mosquitoes, they suck the blood, and they give you a reaction, with more hives, larger ones, once they inject the poison ¿no? And it causes stinging. I squeeze it [the hive] to get the poison out and that’s how it disappears” (mason, 31 years-old).

Ticks, fleas, “*mosca chiclera*”, horseflies, mosquitoes, and triatomines are perceived to acquire *poison* by sucking blood from other animals that “have something”, which was not named or explained. Participants considered that animals carry and attract these insects.

Biomedical terms (*virus*, *parasite*, and *microbe*), popular terms, and metaphors (*venom*, *infection*, and *poison*) were used to associate insects to health problems. Contact through fluids and substances between insects and body-blood-skin is a core concept to construct meaning:

F2: “Because when it [the bug] bites us, it absorbs the blood ¿no?, and hence I understand that in that way it is also a transmitter, although it also occurs via the eggs, and blood is the vehicle ¿no? or the “oil” (housewife, 37 years-old).

The insect´s bite is strongly associated as the principal mechanism through which insects “leave disease” in the human. The population uses the metaphors of a “needle” or a “syringe” to compare to the insect, which causes a piercing sting puncturing the skin and exposing the body and blood to unknown and alien elements. This categorization is also used for triatomines, reinforcing the “mosquito-related” image for all hematophagous insects. A few participants stated that the triatomine faeces carries the disease, although the concept was unclear:

F3: “Well, some people say, I am not sure if it is correct, that as it bites and drinks blood, it also defecates, and that is what infects you, it is what harms you, I am not sure if it is the bite or the fecal material, or I don’t know” (housewife, 37 years old).

Insect bites and skin problems from insects are treated with homemade remedies although the majority of persons “just wait until they are gone”. Traditionally, remedies are made from autochthonous plants and creams, or ointments are applied at home to reduce symptoms, as observed for mosquito, horsefly bites, and for *chinchoma* (houses 09/03/2011 and 27/06/2012; Fig 4D). Domestic treatment for these bites reflects their “normalcy”, and the lack of awareness by the population of the broader threat which triatomine contact represents. Inhabitants expressed agreement to seek care at the local clinic if any family members had a skin reaction like the *chinchoma*, even though most did not know if they would recognize it. One participant affirmed that:

M3: “Nobody really goes to the clinic for an insect bite, not even for scorpions, just for a snake bite, you do really go running [to the clinic]” (farmer, 52 years-old).

### Housing materials, lot arrangement, and gender

**Fig 5:**
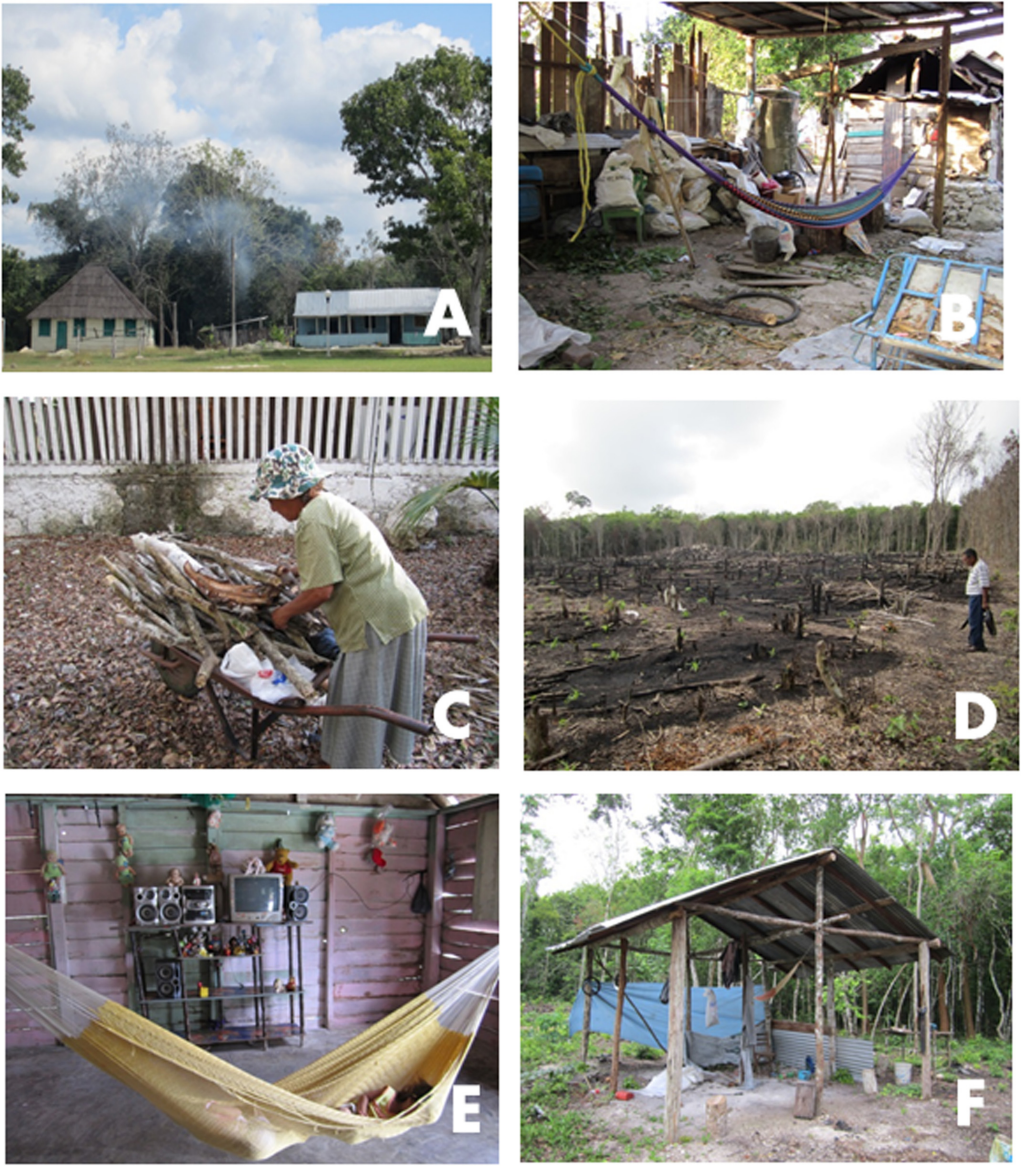
Images of social appropriation of the Zoh-Laguna landscape. A: Wooden houses from the community periphery, surrounded by vegetation. B: Peridomicile structure to store harvest and work tools, with a hammock for siestas. C: Firewood collection. D: Newly planted milpa, on the edge of the *acahual*. E: Children sleep without a bednet during the dry season. F: Structures in the *acahual* including a hammock for resting.

The wood siding on houses provides ample spaces for insects to traverse walls, providing opportunity for interaction with inhabitants. Rustic houses are considered to offer sufficient protection against insects and other natural elements, even though wood houses are perceived to be predisposed to triatomine infestation (Fig 5A). Several women believe:

F4: “Well they (bugs) adapt to a new way of life and when they don’t have their habitat, the most similar is a house, because of the wood” (housewife, 33 years-old);

F5: “Wood is used by insects to reproduce” (housewife, 35 years-old).

Since wooden houses are also perceived as more permeable to insects, the inhabitants believe that this also causes reduced insecticide efficacy:

F6: “Because there [concrete house] you can fumigate and all is closed, but on the other hand, in a wooden house no, the insecticide escapes” (housewife, 35 years-old).

Although there is great care in floor cleaning within houses, walls are not dusted and airing-out or dusting furniture or wall hangings was not observed (Fig 4E). Women are considered (by both men and women) the primary house-keepers, and are responsible for cleanliness inside the house, an important aim of their role. For both women and men, the ideal concept of a “healthy house” is one constructed of cement and brick, maintained clean, well-ventilated, well-illuminated, and that does not offer resting sites for insects. Men are in charge of order and cleanliness in the peridomicile, even though women also participate. Peridomicile cleaning focuses on eliminating inorganic garbage and cutting undergrowth. Undergrowth (*monte*) and dark spaces are considered “dirty” (Table 1). The peridomicile often contains accumulated construction materials (stone, blocks, and piles of sand and soil), tool sheds, and storage products none of which are cleaned or shifted regularly to avoid insects and pests; these may remain unmoved for months to years. Rustic structures usually having thatch or laminated roofs (pressboard or zinc) and wooden walls are used within each property to store construction materials (Fig 5B).

Lack of cleaning and hygienic conditions, dirt, presence of trash, and maintenance of animals outside the house were all mentioned to be associated with biting insects and triatomines inside houses. Despite this perceived interaction, animal pens, poultry yards, and animal sleeping shelters are placed close to houses, and cleaning or clearing practices in these spaces were not mentioned nor observed. Maintenance practices for chickens, pigs, sheep, songbirds, parrots, dogs, and cats were variable depending on the family´s diet and preferences for pets. Chickens, pigs, and sheep are usually maintained close to houses to avoid theft or being hunted by other animals such as fox and wild cats. Nesting hens are maintained in chicken coops located in the garden area and rarely inside houses. Dogs wander freely and rest in the peridomicile where they can find shade, while parrots are kept in cages inside and surrounding houses. Only a few households raise wild turkeys and pigs, highly valued for their taste.

### Increase in landscape modifications and inter-habitat movement in the dry season

**Fig 6:**
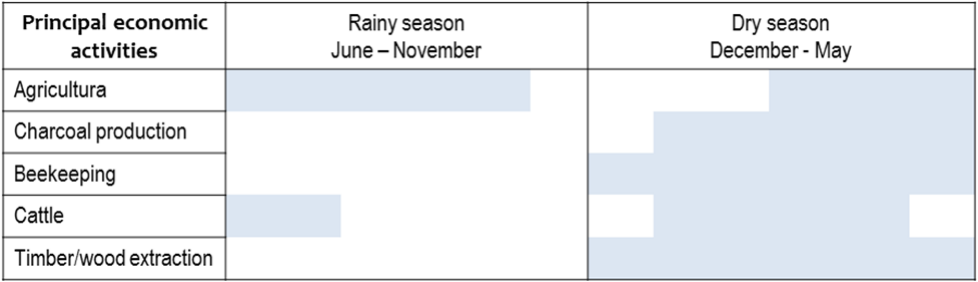
Seasonal dynamics of the principal productive activities in the Zoh-Laguna landscape. Highest intensity and frequency related to productive activities occurs during the dry season. Grey sections indicate temporality of major activities.

**Fig 7:**
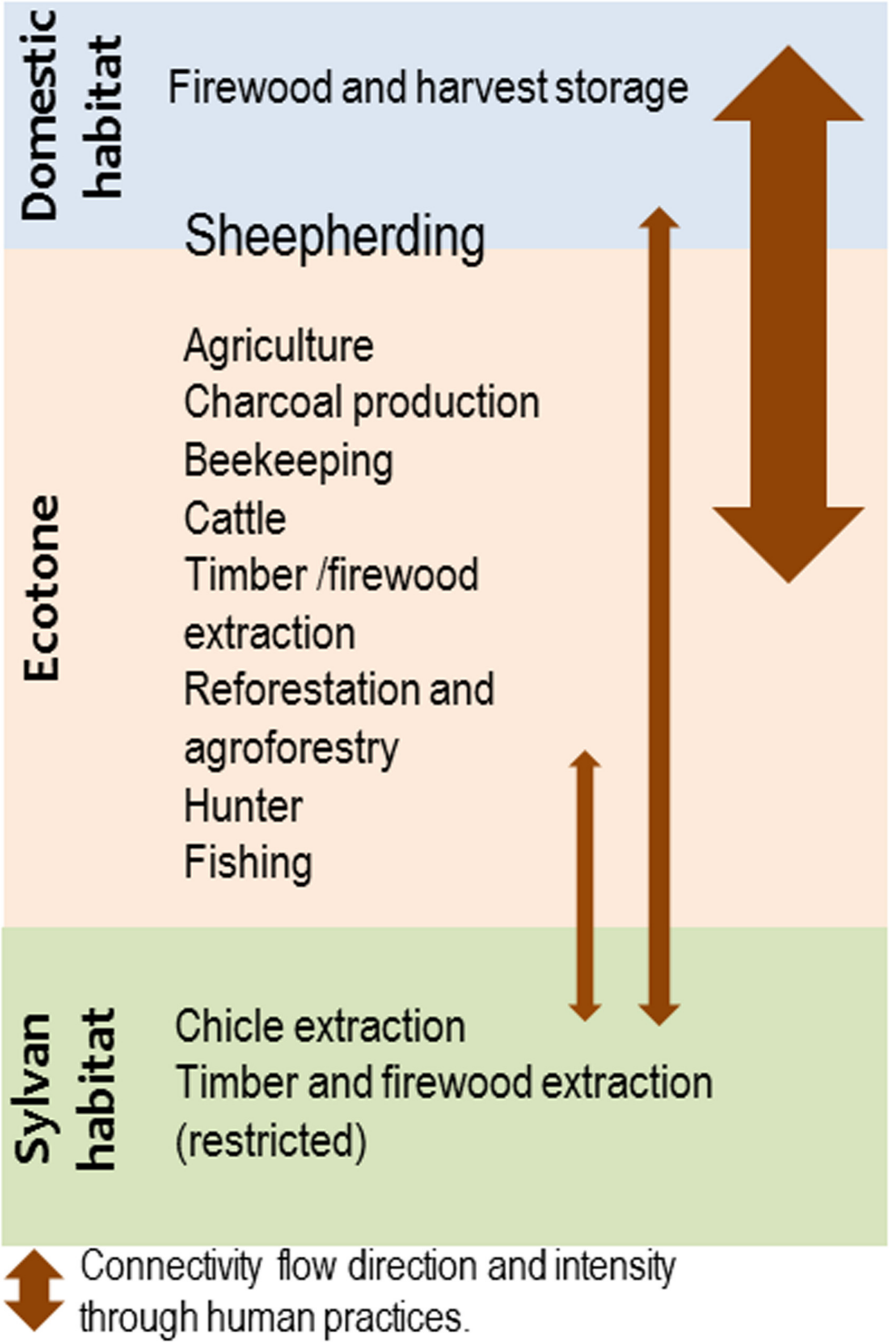
Spatial dynamics of modification and land use social practices in the Zoh-Laguna landscape. Flow direction and size denotes intensity of human practices, greatest between the ecotone and domestic habitats. Intensified *acahual* use is a current strategy to limit the agricultural expansion into conserved sylvatic areas.

Seasonality is a central variable dominating human interaction with livestock, agriculture and natural products within and between landscape fragments. Inhabitants and products are moved either by foot, horse, or vehicle. The human population has little interaction with non-domestic fragments of the landscape in the rainy season since wet calcareous soil is highly impenetrable (Fig 6). There is significantly more intense, diverse, and frequent movement within the landscape in the dry season, particularly for practices carried out between domestic and *acahual* (ecotone) habitats (Fig 7). Households with members working in agricultural activities, commonly but not exclusively associated with land tenure status, store and maintain objects and products such as wood, stone, firewood, and charcoal sacks from the *monte* in the peridomicile for varying periods in the dry season (Fig 5C). The slash and burn agricultural cycle begins during the dry season to plant in time for the initiation of the rainy season (Fig 5D). Charcoal production only occurs in the dry season, when wood for construction, cooking, and charcoal are also collected and stored.

In social imagery, when the *monte* is cut, insects move to the human community and into houses for refuge and resources, while larger wildlife moves to *la montaña* (sylvatic habitat) (Table 1). According to the *ejidatarios*, “there is still a lot of tall *monte*” in sylvatic areas where wildlife can find food and water (Table 1).

### Triatomines associated with humidity-darkness-monte

Participants related that insects “have their season” and “rainy is ideal for them” (accountant, 58 years-old). These observations are based on the local concept that humidity and aquatic breeding ponds are essential for all important insects, including triatomines:

M4: “.....we saw bugs in some rocks at the edge of the pond, that’s why I told you that the bug [the triatomine] looks for water, for the humidity, and because it was already dark, at night you can hear, you can feel how those insects are around you, they come flying, because their wings sound almost like a dragonfly. We turn off the lamplights, and we saw that it was *the Chagas*, but so many, so many of those bugs” (farmer and hunter, 52 years-old).

A local belief is that bugs “like” darkness, interpreted from the fact that they “come out at night”. Most mammal hosts are nocturnal and forage near water sources where there is greatest food abundance. All insects, including triatomines, are considered to originate in the *monte* and not from inside houses, even those found in domestic areas (Table 1):

F7: “From which other place do they [triatomines] come from, if not from the *monte*? All insects come from there” (housewife, 35 years-old).

Popular knowledge about mosquitoes and insectivorous and phytophagous bugs and other crop pests are applied to triatomines:

F8: “Besides to drink blood, they [triatomines] also eat leaves from trees ¿don´t they? Or maybe they eat some small insects” (housewife, 35 years-old).

The inhabitants consider that their interactions with insects are accidental, undesirable, or a result of neglect. Although their first reaction is to kill or drive away the insects, they indicate that children cannot do the same or may even play with them. Women are “disgusted” and frightened of cockroaches, bugs, snakes, and scorpions. Participants had killed triatomines in their houses, or mentioned they would kill them if found in the house, and only a few agreed to catch them in containers and hand them over to the clinic.

House-cleaning and insecticide use are the principal methods used to avoid contact with insects inside houses. Some participants affirm that thorough domestic cleaning is insufficient to keep insects away, because environmental conditions and the rural way of life are primary determinants for insect presence, and hence insect control is not possible if dependent on their actions alone. In the domestic habitat, the population asserts that high humidity and the presence of *monte* explain the presence of bugs. Houses located on the periphery of the town are perceived to have greater risk to become infested with them. When a participant was questioned about why a triatomine could infest his house he said:

M5: “Well, because of the undergrowth, for the *monte*. Yes, because as we are surrounded by *monte*, although we say we are in a town, there is *monte* everywhere, and from there is where triatomines came up or they reproduce” (farmer, 52 years-old).

### Sleep and protection practices in the entire landscape

Zoh-Laguna residents sleep in hammocks or beds inside their houses. During the day, children take naps mostly inside, and during the hottest part of the dry season, men rest in hammocks under shade surrounding houses (Fig 5E). Bednets are used, by the majority of families, principally for children, but also by women (Fig 4F). Far less men than women sleep under bed or hammock nets. Refusal to use nets is due to the higher temperature and lack of air exchange under the net. Men use hammock nets when sleeping in ecotone areas, but only during the rainy season (to prevent mosquito bites).

Insect contact prevention is considered important mostly inside houses, at night, and in the rainy season. Inhabitants believe that mosquitoes and triatomines bite while the inhabitants sleep, since they are unaware of insects at that time. Diurnal sleep periods in open spaces, such as the peridomicile or farm fields and *monte* are not considered a hazard for exposure to triatomines. Farmers, lumberjacks, hunters, and charcoal producers, principally men, stay overnight or take noonday naps in precarious roofed areas in the *monte* (Fig 5F). Since there are fewer mosquitoes in the dry season, there is no perception of risk while sleeping outdoors unprotected in that season. Outside houses, especially in the rainy season, controlling contact with insects is considered to be futile and not within their reach:

M6: “I have gone to the countryside, I am not a farmer but I have been there, and you get covered with mosquitoes, but badly, they are quite abundant […] and blood pours out of everywhere [in the body]. It’s really terrible and there is no effective protection, one cannot say “I put my mosquito-net over me and then I go to countryside” (employee, 58 years old).

M7: “Well now [dry season], in the *monte* there aren’t [mosquitoes], automatically, now you can lay down wherever, there’s no mosquitoes, but in contrast, in the homes, they [mosquitoes] are always there, no matter how careful you are” (farmer and electrician, 41 years old).

Generally, in order to prevent insect and triatomine bites apart from bednets, the population reports and were observed using aerosol insecticides, mosquito coils, and plug-in mosquito repellent. These methods are purchased in local stores.

## Discussion

Reduction of VBT*Tc* has been historically achieved via massive domestic insecticide spray control campaigns, where two invasive and uniquely domesticated triatomine populations were dominant: *Triatoma infestans* in the Southern Cone of South America and *Rhodnius prolixus* in Central America [54–57]. However, since there are no other triatomine species known solely from human habitats, vector exposure reduction for other species in the continent will need to target both hazard and social vulnerability over the entire landscape [58]. Reducing human risk long-term in all exposure areas requires comprehension of the interaction dynamics among vector, parasite, reservoirs, and humans, the latter based on their sociocultural, economic, political, historical, and ecological characteristics. Analysis of social representations and practices which identify these interactions, complement the study of the parasite´s landscape ecology by exploring and analyzing social beliefs, values, knowledge, and human practices related to biological and ecological phenomena, which generate human vulnerability for VBT*Tc* (Table 4).

**Table 4:** Vulnerability components registered in the Zoh-Laguna landscape.

		Domestic	Ecotone	Sylvatic
**Vulnerability from health, disease and clinical care processes**	**Habitat specific**	There is an increase in sleeping practices in the peridomicile in the dry season	Sleep practices increase in the dry season for variable periods (days)	No sleep practice by humans, although hunting practices imply contact due to reduced movement
		Bednets and canister insecticides are used principally in the rainy season	Insect bites tolerated in the rainy season tolerated but exposure periods reduced	No protection measures used
		Women are the overseers of the family health and practices	Men are responsible for their exposure and health risks	
	** **	Bugs easily establish large colonies and may also be moved from the monte	Bugs have small colonies, concentrated near reservoirs (and in turn water)	
	**Habitat common**	Normalization” of insect bites, no care-seeking activity for bug bites		
		There is a strong association of insects with humidity/rain, and a general perception of increased abundance of all nuisance insects in the rainy season;		
		Lack of practices to avoid insect bites in the dry season;		
		Domestic remedies for insect bites.		
	** **	Lack of awareness of triatomines as a disease vector, and of the association with the *chinchoma*		
**Vulnerability from land appropriation and use**	**Habitat specific**	Almost complete modification of vegetation and fauna, with introduction and permanence of humans, livestock, and pets	Constant modification and use, principally in dry season,	Little modification, passive use (hunting, plant collection, firewood collection), no introduced or alternative reservoirs
		Introduction of materials, products from the ecotone and sylvatic during the dry season	Movement of humans to ecotone daily in dry season, and movement of products to domestic areas	Movement of humans occasionally in dry season from domestic to sylvatic
		Presence of livestock and invasive wildlife permanent	Addition of livestock and temporary presence of pets, reduction of wildlife	No permanent livestock or pets introduced
	** **	Houses constructed with different materials, those with wood considered more permeable to insects; cleaning practices limited to floors	No protected sleeping structures or use of hammock-nets; sleep practices in dry season	No human structures, humans sporadically while waiting in hunting blinds
	**Habitat common**	Perception that triatomine bites occur at night and not daytime, and are more abundant in the rainy season		

Few transmission ecology studies have explored potential interactions between inhabitants and triatomines in different fragments of the landscape, since previous and current control programs make the assumption that the majority if not all vector transmission occurs inside houses or domestic spaces [55, 57]. Although evidence for the vast majority of vectors or landscapes does not support this assumption, a handful of studies have analyzed VBT*Tc* in domestic vs. peri-urban areas, referring to the latter as sylvatic (even though they are not conserved fragments). These studies demonstrate that most landscapes are fragmented, with patches having different degrees of modification, and different degrees of continuous or temporal human interaction. Current evidence, in Mexico at least, indicates that humans have contact with bugs not only in domestic spaces, but to differing degrees, in other landscape fragments including conserved patches [1, 59, 60]. Hence, understanding social representations and practices of inhabitants which reveal not only landscape use, but more importantly structural determinants and motives associated with each fragment type, is the first step to identify and understand human vulnerability, and construct socially, culturally and ecologically appropriate, acceptable, and sustainable VBT*Tc* surveillance, prevention, and control interventions.

### The ethno-ecology of vector-borne transmission of *Trypanosoma cruzi*

Social representations associated with VBT*Tc* in Zoh-Laguna are heterogeneous and integrate different types of knowledge, popular concepts, beliefs, and biomedical or environmental information. These are interwoven and people syncretize meanings from their sociocultural perception of the world, producing new hybrid understanding [25]. This heterogeneity is linked to personal experiences with the vector (or other insects), gender roles, variable contact with biomedical information (healthcare personnel), the landscape, livelihoods, and historical circumstances. The impact of exposure to biomedical information is difficult to assess in a population while research is conducted, since the presence, observation, and participation of or with a research group (dedicated to a single topic) and their activities, informal interchange on personal and professional levels, and formal interchange individually or in groups, actively or passively transfer images and messages regarding the vector (wearing uniforms, vehicles, image cards, and pamphlets with the bug image, presence working in the landscape night and day). Awareness of the vector takes place passively, or even though inhabitants are not familiar with its role in pathogen transmission: “… if people from other places come here to catch it [the triatomine], it is because for some reason it is important” (male food-shop owner, 42 years-old).

In contrast to the present results, Rosecrans et al. (2014) analyzing perceptions from the northern Yucatan, found triatomines to be the second most frequently mentioned insect [30]. That study was developed in communities where multiple previous studies had been conducted on CD and hence the proximal importance of the bugs may be due to repeated outside interest [30]. Concepts and social knowledge about triatomines as a disease vector in Zoh-Laguna have been extrapolated from that existing for other harmful insects. This is observed by the association between ecosystem use and modification, and the domestic presence of triatomines: deforestation forces animals “to invade human spaces”, houses on the periphery of the community “are more likely to be infested”, “*monte* is where triatomines live”. According to these social images, the hazard from triatomines originates exclusively from outside the community, and not that bugs have always existed in the habitat. This could indicate a weak association between domestic factors and human domestic practices with infestation. There are many examples of close historical interactions between harmful insects and inhabitants in the Yucatan Peninsula, and most of the diseases continue to be endemic (cutaneous leishmanioses, rickettsial disease, dengue, other arbovirus) or are emerging problems which have not been analyzed from the perspective of social representations. Economic and cultural factors compete and antagonize the individual´s perceived incapacity to control medically-important insects and an association between domestic triatomines and family or community health status is weak. This is probably due to the lack of recognized contact with the vector in domestic spaces, or the fact that recognition of a link between an insect and a chronic disease is difficult.

Extrapolation of information about harmful insects, although imprecise, is a process to anchor knowledge and express attitudes and opinions related to triatomines and vector transmission of the parasite [42, 43]. Part of the population has a vast knowledge of insects with which they coexist in the landscape and of those that they perceive to be dangerous to human health. Social representations about insects harmful to human health, however, open the door to “normalize” the triatomine presence due to daily contact and coexistence in all habitats. A specific example is the perception that humidity and rain is important for triatomine abundance (as for mosquitoes), and as a result, the population protects against all insects including triatomines in the rainy season, and uses no protection when in fact there is greatest exposure to bugs in the dry season. Hence, ecological and biomedical information need to be integrated with popular perceptions to construct a more solid information base on which prevention methods can be explored.

The population uses bed or hammock nets extensively to reduce exposure to insect vectors, at night, but mainly in the rainy season. This is a quite different practice than other communities in the Yucatan Peninsula, where this barrier method is not even mentioned to prevent insect bites [30]. It is an important community practice which should be further developed using long-term insecticide impregnated bed or hammock nets or even perhaps indoor wall linings, for both mosquito and triatomine control [39]. Men use bednets the least at night, and almost never use hammock nets for noon-day siestas outside houses, or in farming areas, much less in the dry season. It is hence necessary to engage men regarding their concepts related to bed or hammock nets in all seasons and during all sleep periods, and in domestic as well as non-domestic areas (while hunting, in farming or livestock activities), and the association of bednet use with family economics and health priorities.

### Disease and *chinchoma* perceptions, and care-seeking practices

Domestic treatment of bites and *chinchomas* are a product of their “normalization”, other family health priorities, contact with other insects, lack of priority by the PHS, and insufficient knowledge integrating their role as pathogen vectors. Since they are not considered important events, rather part of daily life, the “normalization” is reinforced by the fact that the dermal effects disappear by themselves with no special attention, and there are no important further immediate symptoms. The lack of importance assigned to *chinchomas* is not surprising since CD is almost completely neglected by the PHS in Mexico, and vector-borne diseases such as malaria and dengue with less disease burden are identified with high priority. *Chinchomas* produced by the *dimidiata* complex species are smaller and the response is more variable in each person, in contrast to that produced by the *phyllosoma* complex species (north of the Isthmus of Tehuantepec) [60]. While some inhabitants of Zoh-Laguna describe a 5cm induration, others report nothing remarkable, even though the contact events had occurred more than 10yrs previous, and hence memory may have affected perception. Local knowledge and concepts about insect bites and dermal consequences participate actively in the population´s understanding of biomedical information about VBT*Tc*, and are linked to curative and preventive practices. The population not only reacts to the biological reality of VBT*Tc*, but they also act based on the social group´s integration of the information, its significance, and their beliefs [25, 42, 43]. These findings should be considered to design social awareness in order to use the *chinchoma* as a sentinel event for early detection of acute infections.

The population´s concepts regarding *allergy* and *poison* and their impact on the normalization of bug bites, are central to anchor the potential harm of triatomines to health. The concept of *allergy* is used by male farmers and those working in grasslands to distance themselves symbolically from the consequences of the bites and weak bodies, also reported in Colombia in reference to rickettsial disease [48]. Their belief that multiple bites “vaccinate” against harmful effects diminishes the threat they represent, and contrasts with biomedical information which emphasizes that repeated exposure is the principal risk factor for infection with *T*. *cruzi* and potentially for disease [25]. The concept of *poison*, representing the etiology of the disease, has been reported from Argentina [31, 35], Venezuela [38], and Bolivia [40]. Despite the terminology, it is interesting that there is the perception that the insect contains a “poison” (parasite) in Zoh-Laguna, and more importantly, that they identify the origin of this from wildlife and domesticated animals. The population recognizes that the same bugs that bite them, also bite wildlife, and thereby acquire the poison. Once again, appropriate social communication could integrate these concepts to construct an integrated knowledge base which focuses on the source of infection and may more effectively create the necessary prevention to manage bug bite.

In Mexico, CD is neglected and little attention has been given to VBT*Tc* prevention, or to diagnose, provide specific clinical care, and treat patients. Academic groups and civil organizations have generated the primary information base regarding CD, while the PHS has ignored solid evidence since the first national epidemiological survey reporting 1.5% seroprevalence in the country [60, 61]. Obviating the epidemiological evidence has provoked both the lack of prevention and control of VBT*Tc*, and little or no appropriate treatment or clinical care for infected individuals or those with chronic symptoms. In many endemic areas of Latin America, many studies have highlighted the fact that the vector is not associated with disease by an exposed population [25]. This is due once again to the almost unique focus on acute mosquito-borne diseases (malaria, dengue), the disease´s chronicity, and multiple other local health priorities [30, 33]. All triatomine control initiatives (Southern Cone, IPCA, Andean Pact, and Amazonia) have been designed and justified based on vector population reduction or elimination only from domestic spaces, even as a prerequisite to patient treatment [62, 63]. Neither the infected patient´s perceptions nor their representations about the disease have been considered or incorporated into CD control program interventions [25]. Generally, national Chagas programs are incorporated into surveillance and prevention arms of ministries and they do not effectively coordinate with clinical healthcare services for a solid clinical care program. This explains the paucity of controlled patient treatment trials, knowledge regarding the source of disease pathology, and the lack of clinical indicators for disease progression or treatment [62, 63]. Ideally, an integrated program will need to create an intercultural information base which focuses both from biomedical and social perspectives on both the disease and timely diagnosis and access to treatment, and the vector for prevention and control of case incidence. Parasite source reduction in humans and reservoirs through anti-parasitic treatment has never been attempted to limit both the impact of clinical disease progression and reduce domestic bug infections, even though this was the focus for more than 50 yrs of intensive and highly successful malaria control in many countries, including Mexico.

### Livelihoods and differential exposure in the landscape

The analytical landscape framework used in this study has given us the opportunity to analyze spatial and seasonal-temporal human practices and related social representations that generate social vulnerability for VBT*Tc* and overlay this information on biological and ecological determinants of *T*. *cruzi* in the landscape. Specific practices of inhabitants, and their movement of products or animals occur to different degrees within and between habitats, according to season. Hunters and *monte* workers of Zoh-Laguna (men) report triatomine bites in ecotone and sylvatic habitats (during daytime naps, camping in the rainforest, and during their hunting practices near ponds), while women and children report bug bites principally in domestic areas. The population uses the ecotone for productive activities such as farming, bee-keeping, livestock pasture and pens, charcoal production, and collection of useful natural products. The inhabitants not only modify the ecotone by reducing wildlife diversity, but they introduce domesticated livestock (susceptible alternative reservoirs with little co-evolved tolerance mechanisms). Men remain for variable periods (often several days and nights) in the ecotone particularly in the dry season, and may have diurnal or less frequent visits in the rainy season. Their exposure to bugs has been documented both by their reports of bug bites and ecotone bug bloodmeal analysis in Zoh Laguna [50]. This risk has been documented elsewhere in the Yucatan peninsula [59, 64], other Mexican states [60, 65], as well as in camps or temporary settlements in Amazonia [66], other areas of Brazil [67], Colombia [68], Ecuador [69], and Bolivia [70]. Vector-human contact in extra-domestic areas in Zoh-Laguna occurs principally but not exclusively in the dry season. In contrast, the permanent presence of the inhabitants, livestock, companion animals, invasive wildlife (house mouse and urban rats), certain autochthonous rodents, didelphids, or bats in the domestic habitat provide ample resources to bugs year-round. Permanent housing, and storage structures where implements, products, or livestock are maintained are not shifted or their pests controlled. Larger livestock are rarely maintained near houses, concentrating animal corrals in the ecotone or on the periphery of the village, which coincides with the perception that houses on the periphery have more bugs, although no significant spatial bias for greater infestation on the periphery has been measured in Zoh-Laguna [50]. These perceptions could be the focus of engagement interventions in which the population´s perceptions and entomological data can be integrated and used to develop proposals for vector reduction both in the center, as well as on the periphery of the village. Although internal house-cleaning of floors is a priority (for greater hygiene surrounding small children), neither the inner nor outer walls of houses are dusted or cleaned (to disturb bugs). Community-based housing improvements targeting walls, do not have to be costly, rather they need to focus on creating an unstable microhabitat, which repels bugs (constant cleaning or sweeping), or impedes their movement across ceilings, walls, or floors.

Forestry activities have historically influenced local family economies in Zoh-Laguna. Access to natural construction materials (wood, stone, and soil), easily available to *ejidatarios*, is less accessible to those who rent land. The cost of construction materials, family economies, and other factors affect the choice and the techniques used for houses. Inhabitants adopt certain lifestyles based on these factors, and use, transform, and interact with the landscape in specific ways which may constitute important vulnerability components [19–21]. Reported contacts between vectors and inhabitants inside houses is continuous throughout the year (permanent domestic resources) even though greatest interaction occurs in the dry season, due to the reduction in sylvatic hosts and a decrease in barrier use (nets) or insect control (insecticides). The peridomestic habitat provides year-round resource availability (grain, food leftovers, livestock feed) for reservoirs (invasive sylvatic or livestock), essential not only for providing an improved survival for bugs (fitness), but also for parasite transmission [1]. As long as human practices provide improved resources, and maintain triatomine interchange between habitats, there may be continuous risk over all landscape fragments.

### Seasons and human activity

The ecosystem, and particularly the climatic reality of the Zoh-Laguna landscape, dominates human activity and parasite-vector-reservoir interactions. Although most sylvatic mammals reproduce in the rainy season, and hence there are more resources for bugs at that time, rainfall and the geological composition of the soil reduces almost to nil human presence in extra-domestic fragments of the landscape in that season. Farmers plant crops prior to and allow livestock to forage in the rainy season to take advantage of the precipitation, but inhabitants do not remain except for very brief supervision periods in the ecotone and have virtually no interaction with sylvatic areas in that season. However, in the dry season, there are fewer sylvatic mammals (affecting principally ecotone areas since there is no forest canopy), but permanent domesticated livestock herds in the ecotone and domestic areas, provide alternative bug food sources directly or indirectly. In Zoh-Laguna, the peak of human presence and productive activities in the ecotone is precisely when bugs need and forage for new blood sources, in the dry season. The greatest interaction occurs in all fragments when the human population, particularly men, is present and use less barriers or control for insects, due to mosquito-based perceptions (control only necessary in the rainy season).

### Triatomine exposure and gender

Men and women have similar seroprevalence for *T*. *cruzi* infection in most controlled seroprevalence studies (specific exceptions do exist), which could imply that exposure to triatomines is random and not sex-related. However, as is the case in Zoh-Laguna, there may be subtle differences in exposure which may be gender and habitat related. Men have greater vulnerability based on occupation in extra-domestic habitats (farmers, bee-keepers, charcoal producers), while women may have greater vulnerability based on their house-keeping role in the domestic habitat (where vector densities are highest and stable year-round). Although the decision to purchase bed or hammock nets is apparently without gender-related control in Zoh-Laguna, their use is driven by the social construction of gender and sociocultural preferences. Men expressed their individual “physical resistance” to insect bites, based on frequency, most common in those who work in the *monte*. Local concepts of masculinity are associated with physical strength and stamina, and may guide their justification that more bites vaccinate them against illness, and hence there is no need for prevention methods. Based on gender roles, most men are clearly more exposed in all fragments of the landscape, as compared to inhabitants who only sleep inside the village, in all seasons, and use a bed or hammock net inside the house.

This study confirms that the Zoh Laguna population is capable of recognizing bugs and sensitive to report contact with them, but their predisposition to act on the perception depends on other aspects determined by gender, such as risk perception, social representations about vectors, disease, and health-disease priorities. Men may prioritize a “masculine” image which implies not mentioning, preventing, or prioritizing reduction of vector contact for fear of being considered “weak”. Gender-related roles reinforce both denial of susceptibility to illness (weakness) by men, and their distance and absence due to public healthcare prevention programs (Prospera). Since women have been institutionally (and socioculturally) assigned the role of health supervisors for children and the family through these government programs, men easily justify a lack of preventive response for their own health. This social role assignment places the burden of domestic exposure for VBT*Tc* principally on women´s activities. In fact, the presence of bugs in the domestic area is principally related to peridomicile activities and what men introduce to the domestic habitat from ecotone and sylvatic fragments. Historically and currently, vector-borne disease control programs in Mexico have placed the burden of VBT*Tc* transmission and its control unequally between men and women, assigning women both the tasks and the blame when control is not achieved. This was definitely the case for malaria control programs and is clearly the outcome of current dengue control. Lack of social responsibility by civil authorities (> 90% men) to solve water provision and drainage problems, key factors for mosquito abundance, are obviated, while PHS messages continue to assign blame on the “house-keepers”. Bug infestation of all spaces used by the population, and human exposure due to activities will persist as long as institutions and society continue to avoid equivalent responsibility by men and women for family and individual health, and no evidence-based gender-related perspective is used for prevention or intervention design.

Livelihoods, social knowledge and practices, gender, local health priorities, landscape interactions, and the governmental neglect of CD are all important determinants of human exposure and vulnerability for VBT*Tc* in Zoh-Laguna. Most of these determinants are the result of a lack of information exchange and knowledge construction between biomedical and social sources. An association between triatomines and a chronic disease does not spontaneously occur within social thinking and unless inserted into the population´s conversations and imagery, will not convert into a social “object” [42, 43]. The infected patient has rarely been the focus of investment (design of prevention and control programs and interventions, development of pharmacological agents, treatment and patient management research, development of biomarkers) or Chagas control programs, even though disease burden and its social impact is due to the disease, and not per se the vector [32]. Mexico´s neglect of Chagas as a disease at all levels promotes and maintains its “social invisibility” and vulnerability to VBT*Tc*.

## Conclusions

The present study was conducted in parallel with a quantitative landscape ecology analysis, which directly measured biological and ecological parameters of *T*. *cruzi* presence and dynamics, in all reservoir groups, including humans. Both quantitative and qualitative methods have allowed us to integrate spatial and temporal components related to the parasite and landscape, habitats, all reservoirs, and the human population´s practices. Despite participant number and qualitative analysis used herein, the results are representative and either complement or substantiate critical quantitative transmission evidence in this community. Other communities may have distinct historical, environmental, sociocultural, or VBT*Tc* risk, but may use the same integrated framework to analyze key risk components, both exposure hazard and vulnerability to design intervention strategies based on evidence. The study design used herein, and together with the complete landscape analysis, is novel, integrated, and has reduced the uncertainty regarding associations assumed by less robust methods. These findings can be used to design intercultural prevention interventions adapted to the population´s context and needs.

## Supporting Information

S1 Table Supplemental information. Original quotes in Spanish.
(DOCX)
