# Supplementary material for: Social Representations and Practices Towards Triatomines and Chagas Disease in Calakmul, México
Source: PLoS One. 2015 Jul 23;10(7):e0132830. doi: 10.1371/journal.pone.0132830 (PMC4512683; doi:10.1371/journal.pone.0132830)
Supplement: S1 Table — Original quotes in Spanish. (DOCX) [file pone.0132830.s001.docx]

Original Quotes in Spanish transcribed from verbal interviews and translated to English and used in text.

F1: “Depende, a veces llegan a durar 2 o 3 días pero cuando uno es alérgico hasta materia le sale.” (Ama de casa, 33 años).

M1: “Pues de nosotros nunca nos hizo daño [el triatomino], nunca, pero pues vuelvo a repetir como uno ya está acostumbrado a piquetes y todo pues… pues no, no te hace nada, pero si no está acostumbrado uno sí lastiman a uno, sí lastiman.” (Campesino, 65 años).

M2: “Pues también como los moscos sacan la sangre pero ahí si te dan, sacan más ronchas, más gruesa pues, una vez que inyecta el veneno digamos ¿no? y provoca picazón. Yo lo exprimo para que salga el veneno y así se desaparece.” (Albañil, 31 años).

F2: “Porque ella [el triatomino] al picarnos es lo que absorbe ¿no? la sangre entonces pues yo entiendo que por ahí es también el transmisor, aunque supuestamente los huevecillos también ahí y pues la sangre es el motor de uno ¿no? Más bien el aceite” (Ama de casa, 37 años).

F3: “Pues, dicen, no sé, si es así, ahora sí que yo así le entiendo o así lo escuché, que es que al picarte te está picando y está haciendo popó, y esa cosa es lo que te infecta, es lo que te hace mal, no sé si es el piquete o es que se está haciendo [popo] o no sé” (Ama de casa, 37 años).

M3: “…realmente nadie va a la clínica por un piquete de insecto, ni por el alacrán, sólo la culebra si vas corriendo.” (Campesino, 52 años).

F4: “Pues ellos se adaptan a nuevas formas de vida y al no tener su hábitat pues lo más parecido a su hábitat pues es una casa, por la madera” (Ama de casa, 33 años).

F5: “la madera sirve para reproducirse” a los insectos (Ama de casa, 35 años).

F6: “Porque ahí [casa de concreto] se puede fumigar y todo se encierra, en cambio, en una casa de madera no, se sale por todos lados” (Ama de casa, 35 años).

M4: “…salimos de cacería un día y nos subimos en unos árboles, en unas piedras allá a la orilla de una aguada, por eso le digo que el bicho busca mucho el agua, por la humedad, pues como está de noche, pues oyes, se sientes como te están andando los bichos estos, es cuando vienen volando, porque suenan sus alas como casi como la libélula. Prendíamos las luces y veíamos que son las Chagas, pero mucho, mucho, muchos de esos bichos.” (Campesino, 52 años).

F7: “¿pues de donde más van a venir sino del monte? Es el procedimiento de otros bichos” (Ama de casa, 35 años).

F8: “A parte de chupar sangre, comen hoja ¿no? Algún otro bichito más chico” (Ama de casa, 35 años).

M5: “Pues por la maleza, el monte. Sí, porque como estamos rodeado de monte pues, aunque decimos que estamos en el pueblo pero por todos lados hay monte, y de ahí mayormente es donde salen o se reproducen ahí [los triatominos]...” (Campesino, 52 años).

M6: “…a mí me ha tocado ir al campo, yo no soy campesino pero me ha tocado ir al campo y cómo se tupe uno de zancudos. Pero así, así bastante pues bastante, de me está uno aquí y aquí, chorrea sangre por todos lados, no, sí es terrible y ahí no hay protección pues de que diga uno “me pongo mi pabellón y me voy al campo” (Empleado, 58 años).

M7: “…ahorita pues en el monte no hay [moscos], automáticamente, ahorita te puedes acostarte donde sea, no hay moscos, y en cambio en la casa sí hay, por muy cuidado que esté pero siempre” (Campesino y electricista, 41 años).
